# Prevalence of self-reported finger deformations and occupational risk factors among professional cooks: a cross-sectional study

**DOI:** 10.1186/1471-2458-11-392

**Published:** 2011-05-26

**Authors:** Miwako Nagasu, Kazuhiro Sakai, Kazutaka Kogi, Akiyoshi Ito, Edith JM Feskens, Shigeru Tomita, Yoshiomi Temmyo, Mitsuo Ueno, Shigeji Miyagi

**Affiliations:** 1 Division of Human Nutrition, Wageningen University, Wageningen, The Netherlands; 2 The Institute for Science of Labour, Kanagawa, Japan; 3 School of Health Sciences, University of Occupational and Environmental Health, Kitakyushu, Japan; 4 School of Medicine, Dokkyo Medical University, Tochigi, Japan; 5 Minatomachi Medical Center, Kanagawa, Japan; 6 Institute of Occupational Safety and Health, All-Japan Prefectural and Municipal Workers' Union, Tokyo, Japan; 7 Department of Health Sciences, Kagawa Nutrition University, Saitama, Japan

## Abstract

**Background:**

Previous studies have pointed out that the school lunch workers in Japan are suffering from work-related disorders including finger deformations. The purpose of this study was to investigate the prevalence of self-reported finger deformations and the association with job-related risk factors.

**Methods:**

A cross-sectional questionnaire study of 5,719 subjects (response rate: 81%, 982 men and 4,737 women) was undertaken during September 2003 to February 2004.

**Results:**

Finger deformations were found among 11.7% of the men and 35.6% of the women studied, with significant differences among sex, age and sex-age groups. For both men and women the pattern of finger deformations across the hand was similar for the right and the left hand. For women, the deformations were found in about 10% of the distal interphalangeal joints of all fingers. Based on multiple logistic regression analyses, the factors female sex, age, the number of cooked lunches per cook and cooking activities were independently associated with the prevalence of finger deformations. High prevalence odds ratios were found for those frequently carrying or using tools by hands such as delivering containers, distributing meals, preparing dishes, washing equipment, cutting and stirring foods.

**Conclusions:**

Among the school lunch workers studied, women had a higher prevalence of finger deformations on all joints of both hands. Various cooking tasks were associated with the prevalence of finger deformations. The results suggest that improvements in working conditions are important for preventing work-related disorders such as finger deformations.

## Background

Hands osteoarthritis is the most common joint disorder in adult population [1-3]. The symptoms include deformations of joints, joint swelling, pains and limitations of motions [4]. Epidemiological studies have reported that the rate of hand osteoarthritis range between 32.6% and 70% [4,5]. Finger deformations are a main symptom of hand osteoarthritis.

Risk factors for finger deformations have been studied, such as genetic factors [6-8], age [9], sex [5,9-12] and occupational factors, including the amount of manual work [10,13,14]. Finger deformations have frequently been reported as work-related disorders among cooks working in school lunch services in Japan, which have high workloads [11,14-17]. Several studies of cooks have reported that the symptoms due to finger deformations are similar to Heberden's nodes and accompany hand osteoarthritis [11,14,17-19]. Occupational risk factors for finger deformations accompanying hand osteoarthritis among professional cooks are likely [10,14,20,21]. Further identification will form the basis of taking comprehensive preventive measures for reducing hand-operated workload and improving kitchen equipment.

Therefore, we carried out a study on a nationally representative sample of cooks. The objectives of the study were (1) to estimate the prevalence of self-reported finger deformations and (2) to indentify the job-related factors that could be associated with the (high) prevalence of self-reported finger deformations.

## Methods

This study was part of a research project about health outcomes and occupational risk factors among professional cooks in Japan [22]. The cross-sectional survey was conducted using a mailed self-administered questionnaire (additional file 1). The purpose was to investigate the prevalence of self-reported finger deformations and to indentify the job-related factors related to the work of cooks.

Participants included professional cooks working in school lunch services of elementary schools. According to the data in a school lunch catalogue [23], school lunches were provided for 7.19 million children in 2002. A total of 52,790 full-time cooks were working in the school lunch services in 2002 [23]. The principal task of these cooks was to provide meals as lunch for all pupils 5 days a week, year round, except during seasonal vacation periods.

The study sample was selected through the head office of the All-Japan Prefectural and Municipal Workers Union. All the 47 prefectures and municipalities were included in our study to obtain a proper representation of cooks engaged in school lunch services all over Japan. The union leaders maintained a list of the cooks working in school lunch services. There are a total of 47 prefectures all over Japan. From one prefecture 500 cooks were selected at random from the list of cooks. Similarly 200 cooks from each of 10 prefectures were selected (total 2000), 300 cooks from each of five prefectures (total 1500), and 100 cooks from each of 31 prefectures (total 3100) were randomly selected. Thus a total of 7100 cooks were selected for this study.

A mailed self-administered questionnaire was used to collect information about finger deformations, demographic characteristics and job-related factors. The questionnaire asked about demographic factors such as age and sex. Information about job-related factors included various aspects of work activities. The question of work activities was as follows: What do you think are the factors that affected your finger joints most? Then subjects selected an answer among work activities' choices. Finger deformation was defined as an experience of an episode or episodes of finger deformation anytime during the duration of employment as a professional cook.

Self-reported questionnaires with a simple line-drawing of fingers have been used successfully for self-reported assessment of physical characteristics [24]. One question referred to finger joints which were deformed on either hand presented on a picture showing 18 joints of both hands: eight distal interphalangeal (DIP) joints, and eight proximal interphalangeal (PIP) joints and two interphalangeal (IP) joints of the first fingers. The subjects were asked as follows: which parts of your fingers are deformed by work activities, excluding congenital malformation of fingers and non work activities of fingers? The participants reported the location by putting a mark on the picture. The questionnaire was adapted from a Japanese edition of a questionnaire used in previous studies [25,26]. This enabled us to compare the results of the present study with those of previous ones.

The cross-sectional survey was carried out from September 2003 to February 2004. This study was supported by the All-Japan Prefectural and Municipal Workers Union. The questionnaire was mailed to 7,100 professional cooks through the branches of the union located all over Japan. The questionnaire form was enclosed in an envelope that also contained an informed consent form. The anonymity and confidentiality of the information to be provided was detailed in the consent form. Completed questionnaire forms were mailed back to the investigators. The study was approved by the Research Ethics Committee of the Institute for Science of Labour in Japan.

Of 7,100 cooks to whom the questionnaire was sent, 6,365 (90%) returned the completed questionnaire forms by mail. In the final analysis, 646 forms were excluded, because information about age and sex of respondents or other important information was incomplete. Therefore the final response rate was 5,719 (81%).

The statistical analysis was carried out using the SPSS 16.0 computer package. The prevalence rates, the prevalence odds ratios and their 95% confidence intervals were calculated. We investigated the adjusted prevalence odds ratios and 95% confidence intervals of finger deformations by logistic regression analysis taking into account sex, age and work activities. Work activities included the number of cooked lunch per a cook, moving containers, distributing meals to food containers, preparation and storing the dishes in containers, washing food containers, delivering milk cases and food containers to classes, washing dishes, stirring foods in a large cooker, washing cookers and kitchen sinks, delivering containers to each class, cutting the ingredients, preparation of the ingredients for cooking, cleaning the floor in the kitchen and washing used spoons and chopsticks.

## Results

The study included a total of 5719 participants, 982 men (17.2%) and 4737 women (82.8%). Mean age of the men (41.4 ± 9.8 years) was significantly lower than that of the women (47.5 ± 9.1 years) (p < 0.001).

The prevalence of finger deformations (FD) was 11.7% among men and 35.6% among women, resulting in a prevalence odds ratio (POR) for sex of 4.17 (95% CI, 3.40-5.11) (Table 1). Age was strongly associated with the prevalence of FD. The differences in the prevalence odds ratios of FD across different age groups were significant both sexes, but larger among female cooks than among male cooks.

**Table 1:** Demographic characteristics of the subjects studied.

	Finger deformations			
	
	n	Prevalence (%)	Prevalence **odds ratio **^**1)**^	95% Confidence interval
Sex				
Male	982	11.7	1	
Female	4,737	35.6	4.17	3.40-5.11
Age				
39 or less	1,393	12.3	1	
40-49	1,757	23.4	2.17	1.79-2.63
50 or more	2,569	47.4	6.40	5.36-7.64
Sex and age				
Men				
39 or less	427	8.0	1	
40-49	332	11.1	1.45	0.89-2.37
50 or more	223	19.7	2.84	1.76-4.69
Women				
39 or less	966	14.3	1	
40-49	1,425	26.2	2.14	1.72-2.65
50 or more	2,346	50.0	6.01	4.93-7.32

1) Prevalence odds ratios were not adjusted.

**Figure 1:**
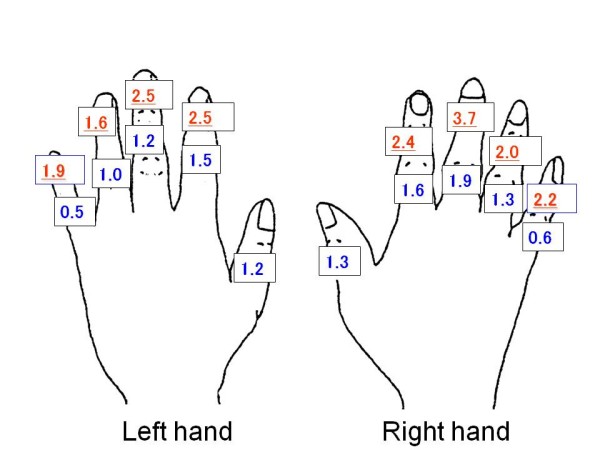
**The prevalence of deformed finger joints (%) (male cooks n = 982)**.

The prevalence of FD in joints and fingers of male cooks is presented in Figure 1. Among both hands, the third distal interphalangeal (DIP) joints (right 3.7%, left 2.5%), the second DIP joints (right 2.4%, left 2.5%), the fifth DIP joints (right 2.2%, left 1.9%) and the fourth DIP joints (right 2.0%, left 1.6%) were affected, followed by the proximal interphalangeal (PIP) joints of the third, second and fourth fingers, IP joints of the first fingers and the joints of the fifth fingers.

**Figure 2:**
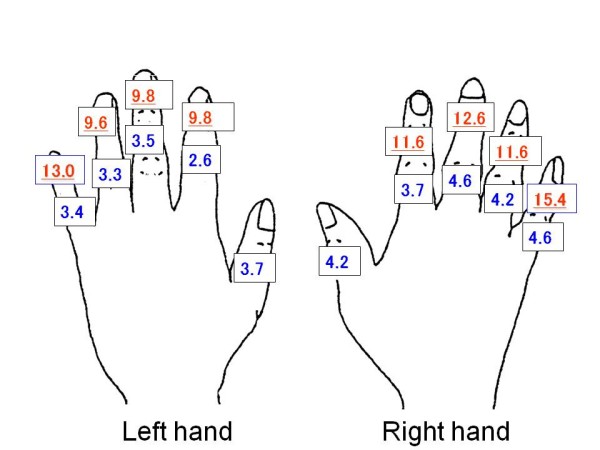
**The prevalence of deformed finger joints (%) (female cooks n = 4,737)**.

The prevalence of FD among female cooks is presented in Figure 2. The fifth DIP joints (right 15.4%, left 13.0%), the third DIP joints (right 12.6%, left 9.8%), the second DIP joints (right 11.6%, left 9.8%) and the fourth DIP joints (right 11.6%, left 9.6%) were frequently involved, in this order, and followed by smaller frequencies at the PIP joints of the fifth, third and fourth fingers, the IP joints of the first fingers and the joints of the second fingers.

Among both sexes the prevalence of FD was more common in the DIP joints than in the PIP joints. Overall, the distribution of FD over the involved joints showed a distinct pattern, comparable in both hands. However, the joints of the right hand showed generally a somewhat higher prevalence rates than those in the left hand, except for the second DIP joint among male cooks.

We investigated sex, age and job-related factors such as contents of work activities as potential risk factors for FD. Mean number of years in occupation was 14.9 yrs (S.D. 9.5) for male cooks and 18.2 yrs (S.D. 8.9) for female cooks. As the Pearson's correlation coefficient of the mean duration of employment and the mean of age was very high (r = 0.696, p < .0001), we used only age in the logistic regression models to avoid problems with multi-collinearity.

The results of multiple logistic regression analyses confirmed the significance of the relationship between job-related factors and the prevalence of FD, as shown in Table 2. For women, the number of cooked lunches per cook was significantly associated with FD. Among contents of work activities, stirring foods in a large cooker, distributing meals to food containers, cutting the ingredients, preparation of the ingredients for cooking, preparing and storing dishes in containers, washing food containers, washing dishes, washing cookers and kitchen sinks, delivering containers to each class and delivering milk cases and food containers to classes were significantly associated with FD. As regards the job-related factors, the highest prevalence odds ratio was found for moving containers by the hands between the kitchen and the classes.

**Table 2:** Adjusted prevalence odds ratios of self-reported finger deformations for selected job-related factors.

	Male		Female	
	**Adjusted prevalence ** **odds ratio **^**1)**^	**95% Confidence ** **interval**	**Adjusted Prevalence ** **odds ratio **^**1)**^	**95% Confidence ** **interval**

Age groups				
39 or less	1		1	
40 - 49	1.34	0.80-2.20	**2.10**	**1.67-2.61**
50 or more	**3.08**	**1.85-5.11**	**6.27**	**5.13-7.68**
Number of cooked lunches per cook				
1 - 149 meals	1		1	
150 - 199 meals	0.41	0.22-0.74	0.99	0.85-1.16
200 meals or more	0.71	0.45-1.14	**1.30**	**1.10-1.54**
Contents of work activities				
Stirring foods in a large cooker	**6.67**	**2.28-19.49**	**3.74**	**2.32-6.03**
Distributing meals to food	**5.53**	**1.62-18.96**	**4.64**	**2.76-7.79**
containers				
Cutting the ingredients	**2.47**	**1.20-5.06**	**2.96**	**2.39-3.66**
Preparation of the ingredients	**2.72**	**1.38-5.35**	**3.32**	**2.61-4.21**
for cooking				
Preparing and storing the	**2.68**	**1.13-6.40**	**4.03**	**2.93-5.54**
dishes in containers				
Washing food containers	**4.40**	**2.02-9.59**	**4.01**	**3.00-5.36**
Washing dishes	**2.21**	**1.17-4.18**	**3.42**	**2.80-4.19**
Washing cookers and	2.78	0.73-10.55	**2.67**	**1.62-4.39**
kitchen sinks				
Delivering containers to	3.88	0.97-15.57	**3.30**	**1.78-6.12**
each class				
Delivering milk cases and food	N.A^2)^	N.A^2)^	**4.27**	**2.36-7.73**
containers to classes				
Cleaning the floor in	N.A^2)^	N.A^2)^	3.98	0.91-17.35
the kitchen				
Moving containers	9.42	0.71-125.02	2.93	0.67-12.87
Washing spoons and	0.65	0.08-5.05	1.02	0.50-2.09
chopsticks				

1) Prevalence odds ratios were adjusted for all items included in the table. Prevalence ratios referred to the following groups: age 39 or less, number of cooked lunches per cook 1~149 and no burden for all contents of work activities.

2) N.A: not applicable

## Discussion

In this cross-sectional study of finger deformations, a high prevalence of self-reported finger deformations (FD) was found among professional cooks working in school lunch services. This prevalence was especially high in women and increased with age. The questionnaire results indicated that the prevalence of FD was associated with several job-related factors.

This study had some strong points. First of all, the study was conducted in a large sample of both male and female cooks selected from all over Japan, with a good response rate of 81%. Secondly, this study could determine the difference in the prevalence of FD between women and men who were engaged as professional cooks in Japanese elementary schools.

However, this study also had certain limitations. As for finger deformations, self-reported prevalence rates obtained by using a questionnaire method may underestimate the prevalence of FD. Self-reported FD reflects the corresponding subjective symptoms among cooks. Previous studies suggest that cooks with painless deformed fingers generally do not mention their symptoms [27]. Therefore, further clinical investigations with x-ray examinations may be needed to reveal FD among cooks [28].

Overall, the prevalence of FD was 11.7% among men and 35.6% among women. The observed prevalence of FD among male cooks was higher than the self-reported prevalence rates (4.9-8.1%) in a nation-wide questionnaire study [16]. As for males, the difference in the prevalence of FD may be related to the higher mean age of the present study (41.4 ± 9.8 years) compared to the previous one (35.5 years ± 9.9 years) [16] and occupational risk factors. The prevalence of FD among female cooks in our study was similar to that of female workers in a paper mill (33.2%) [29,30]. It is possible that there is similarity in the characteristics of the occupational activities, since both cooking and working in a paper mill involves using fingers excessively with repetition of strong and precise pinching movements and gripping heavy materials.

The prevalence of FD among women was significantly higher than among men in this study. Some previous studies reported that Heberden's nodes and hand osteoarthritis with finger deformations were overwhelmingly most common in females [4,5,12,31-33]. It is possible that there are differences between men and women in the presence of risk factors and protective factors. Previous studies suggested that hormonal, genetic and occupational aspects could have played a role [8,34,35]. Hand OA mostly occurs among women at the time of menopause and one possible explanation is that women may have inherently weaker joint cartilage than men. However, the influence of hormonal and genetic factors in the development of hand OA is controversial among both men and women [8,35]. Our findings suggest the need for further investigation of the role of gender in the same occupation.

Increasing age was significantly associated with the prevalence of observed FD in this study. A surprising finding was that young age groups also frequently reported finger deformations. Some studies reported that the rate of Heberden's nodes tend to increase with age, and that people in third and old age groups suffer more frequently from finger deformations than younger age groups [3,5,11,36,37]. A previous study reported that the prevalence of FD among those of age less than 50 was very low and Heberden's nodes occurred as an age-related degenerative change [10]. Kalichman et al. [3] indicated that age was the most effective factor in determining both the frequency and severity of hand osteoarthritis. In the current study, the observed prevalence of FD among female cooks (14.3% and 26.2% among those in their 30 s and 40 s, respectively) was higher than that in the general population (10.6% and 20.7% among those in their 30 s and 40 s, respectively) [12]. According to the results of our study, we may conclude that the prevalence rate among cooks increases with age and with their manual workload as professional cooks.

The result of this study also revealed the prevalence of self-reported FD on the right hand was somewhat higher than on the left hand. This has been shown before [36-39]. Most of workers (94% to 97.3% of the subjects in previous studies) were right-handed [36-39]. Acheson et al. [38] reported a high prevalence of osteoarthritis in the right hand when compared with the left hand in right handed persons. Wilder et al. [37] also reported the same result except for the first carpometacarpal joints. Professional cooking work in school lunch services is one of the few occupations that involve extensive manual work. These cooks perform their work with the dominant hand mainly by cutting a lot of ingredients with a knife, while mixing motions with a spatula and various other motions. In addition to using the dominant hand, cooks need to use the non-dominant hand to carry heavy containers and to use large tools. Consequently, the left hand also showed high prevalence of FD. Both one-sided and two-sided manual work and too heavy workload may be associated with finger deformations.

For both men and women, the prevalence of FD in the DIP joints was higher than that in the PIP joints on both hands. Also previous studies reported that the DIP joints were the most frequently involved joints, with a higher FD prevalence in both hands compared to PIP joints in both hands [16,28-30,37,39]. In the present study, the pattern of the involved joints of the right and the left hand were symmetrical. For men, finger deformations were found in the DIP joints of the third, the second, and the fifth fingers in both hands. For women, the DIP joints of the fifth, the third, the second and the fourth fingers in both hands were most frequently involved in this order. Some previous studies reported that the highest prevalence was found in the joints of the fifth finger [10,39]. As regards the results of our study, it is likely that male cooks grasp objects using the third and the second fingers. However, female cooks use more the fifth and the third fingers. The impact of grip motions and the difference of the hand size between male and female cooks may affect the location of the deformity

Our result of DIP joints being more involved than PIP joints suggest that both anatomic and functional differences exist [40]. The repetitive pinch and grip motions near the fingertips are associated with the development of finger deformations in most DIP joints [18]. As for professional cooks, they use larger and heavier utensils for preparing a large number of lunches for students than in the case of household cooking. Caspi and co-workers reported that housekeeping load did not influence OA [35]. Therefore, our results suggested that cooking activities as a professional cook using special utensils frequently involved the fingertips such as DIP joints by pinch and grip motions.

We also investigated whether specific hand workloads were associated with finger deformations. The results of the logistic regression analyses confirm the significance of the relationship between many activities of these job-related factors and the prevalence of FD. Positive associations were found, mainly those with repetitive cooking activities. There were two main kinds of motion categories that were shown to be involved. One was to grip the handle of heavy utensils or containers, distributing meals to food containers and delivering milk cases and food containers to classes. The other was to pinch cooking utensils and many plates by fingertips tightly as in the case of preparing and storing dishes in a container, washing food containers, washing dishes, stirring foods in a large cooker, washing cookers and kitchen sinks, cutting ingredients and preparation of ingredients for cooking. This emphasizes the importance of general heavy tasks (to grip the handle and carry heavy staff using both hands) and specific hand tasks (to pinch and use utensils by fingers on a dominant hand) in the development of hand FD. Thus, specific cooking workloads may play a role in FD.

The result of previous studies indicated that it is possible to develop preventive strategies such as the prevention of the excessive use of the finger joints at work. An example may be the provision of an appropriate handle for a manual worker to carry heavy materials by means of pinch and grip motions [10,41]. Hadler et al. [42] and Kido [29] reported that hand osteoarthritis and finger deformations developed in joints overused among female workers in textile and paper mills. The finger activities in textile and paper mills are similar to cooking activities done by professional cooks. Their jobs also involve repetitive pincer grip motions. The first and other fingers are pressed together at their tips to hold objects. Sometimes they carry heavy materials and hold the handle of containers. Appropriate handles and the use of ergonomic design can reduce exposure to cumulative trauma [43]. The tendency to flexion deformity of the fingers could be prevented by means of decreasing manual tasks and, if necessary, by the purchase of new kitchen equipment.

## Conclusions

Our study indicates that there is a clear association between finger deformations and job-related factors among Japanese cooks. The self-reported prevalence of FD was high. This high prevalence was also significantly associated with demographic characteristics. The results also indicate the potential of taking comprehensive preventive measures. For reducing the prevalence of FD among cooks, it is generally necessary to address a range of work factors. Of particular importance may be reducing the daily hand-operated workloads per person and improving kitchen equipment such as tableware washing machines and handles of containers, as well as kitchen tasks such as using push carts instead of carrying heavy containers by the hands in the kitchen. The need to develop occupational training programs for school managers and cooks aimed at the prevention of work-related disorders such as FD should be highlighted.

## Competing interests

The authors declare that they have no competing interests.

## Authors' contributions

MN was the principal investigator, participated in the design and protocol preparation, participated in the data collection, data analysis and interpretation of results, and wrote and revised initial drafts of the manuscript. KS assisted in design and protocol preparation, and made a substantial contribution toward analysis, drafting the manuscript, and correcting the initial and final drafts of the manuscript. KK provided advice, technical assistance and revised the manuscript. AI assisted in design and protocol preparation, made a substantial contribution to the analysis, drafting of manuscript and correcting the initial and final drafts of the manuscript. EF provided advice, technical assistance and revised the manuscript. ST provided assistance with the analysis and manuscript preparation. YT contributed to the protocol, and helped with the design of the study and manuscript preparation. MU helped with the design, protocol, and data collection. SM conceived the study, set up the design, guided and participated in the analysis, and revised the final manuscript. All authors read and approved the final manuscript for submission for publication.

## Pre-publication history

The pre-publication history for this paper can be accessed here:

http://www.biomedcentral.com/1471-2458/11/392/prepub

## Supplementary Material

Additional file 1 **Health Questionnaire for Cooks in Lunch Services**. This questionnaire includes the questions which were used in the manuscript only. Click here for file
