# Supplementary material for: Prevalence of self-reported finger deformations and occupational risk factors among professional cooks: a cross-sectional study
Source: BMC Public Health. 2011 May 26;11:392. doi: 10.1186/1471-2458-11-392 (PMC3126742; doi:10.1186/1471-2458-11-392)
Supplement: Additional file 1 — Health Questionnaire for Cooks in Lunch Services. This questionnaire includes the questions which were used in the manuscript only. [file 1471-2458-11-392-S1.DOC]

Health Questionnaire for Cooks in Lunch Services

Note: This questionnaire includes the questions which were used in the manuscript only.

**《 About yourself 》**

Q 1.　Sex：　1. Female 2. Male

Q 2.　Age：　1. Under 25 years old 　2. 25～29 years old　 3. 30～34 years old　 4. 35～39 years old

5. 40～44 years old 　6. 45～49 years old　 7. 50～54 years old 　8. 55～59 years old

　 9. Over 60 years old

**《 About your work 》**

Q 8.　When did you get the job as a cook in school lunch service?　（　　　）Year（　　　　）Month

Q 11. How many meals do you cook in your workplace? （all pupils and staffs）　　（　　　　　　　　）meals

Q 13. How many cooks work in your workplace? 　　（　　　　　　　　）People

**《 Fingers 》**

Q 60. Do you have any part of finger which has bent or deformed currently?

　　1. No　 2. Yes

Q 62. Which parts of your fingers are deformed by work activities with your hands, excluding congenital malformation and non work activities of fingers? Please indicate all parts on the numbers? (multiple answer）


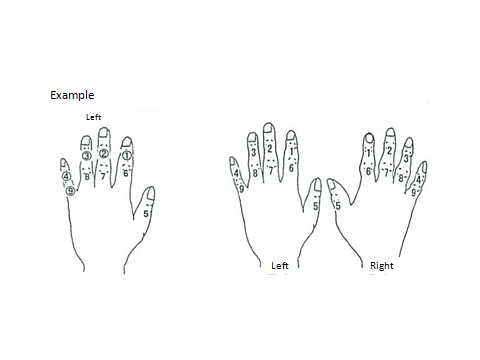


Q 65. What do you think are the factors that affected your finger joints most?

1. Preparation of the ingredients for cooking 2. Cutting the ingredients 3. Stirring foods in a large cooker 4. Distributing meals to food containers 5. Washing cookers and kitchen sinks 6. Delivering containers to each class 7. Washing food containers 8. Washing dishes 9. Washing spoons and chopsticks 10. Preparing and storing the dishes in containers 11. Cleaning the floor in the kitchen 　12. Moving containers 13. Delivering milk cases and food containers to classes 14. non-occupational reason 15. I don’t know 16. Others（　　　　　　　　　　　　）

　Answer ( )
